# Evaluation of definitive histopathological results of patients diagnosed with endometrial polyps: a tertiary care center experience

**DOI:** 10.4314/ahs.v22i1.16

**Published:** 2022-03

**Authors:** Reyhan Gündüz, Elif Ağaçayak, Gülcan Okutucu, Ulaş Alabalik, Mehmet Sıddık Evsen

**Affiliations:** 1 Dicle University, Faculty of Medicine, Department of Obstetrics and Gynecology/Diyarbakır/Turkey; 2 Dicle University, Faculty of Medicine, Department of Pathology/Diyarbakır/Turkey

**Keywords:** Endometrial polyp, premalignancy, malignancy, hysteroscopy, hysterectomy, endometrial sampling

## Abstract

**Background:**

Although endometrial polyps are generally benign, there are also risks of malignancy.

**Objectives:**

To determine the premalignancy and malignancy prevalence in patients diagnosed with endometrial polyps and to investigate factors affecting premalignancy and malignancy.

**Methods:**

In our retrospective study, patients who were diagnosed with endometrial polyp with endometrial samples and who underwent polypectomy by hysteroscopy or hysterectomy within one year were included.

**Results:**

Premalignant / malignant histopathological results were detected in 7 (2.8%) patients. There were no statistically significant differences in histopathological results and endometrial sampling indications between premenopausal and postmenopausal patients. Hysterectomy in patients with premalignant/ malignant results and hysteroscopy in patients with benign results were found to be significantly different. There was not a statistically significant difference between patients with benign results and those with premalignant/malignant results in menopausal status, symptoms, status of hormone replacement therapy and endometrial polyp size.

**Conclusion:**

The possibility of premalignant/ malignant results in patients diagnosed with endometrial polyps should be kept in mind. The menopausal status, symptoms, sizes of endometrial polyps and whether or not the patient is on hormone replacement therapy should be considered while making the management plan. However, these should not be the decisive factors on their own.

## Introduction

Endometrial polyp is defined as the excess hyperplastic growth of the endometrial gland and stroma that protrudes from the surface of the endometrium. They can be asymptomatic. Most of the endometrial polyps are benign; however, malignancy can also be seen in some women.^1^

There is very little data in the literature about endometrial polyps, some of which can be asymptomatic. For this reason, it is hard to determine their prevalence. The prevalence of clinically recognized polyps increases with age and is observed to be higher in postmenopausal women than premenopausal women. 2

Endometrial polyps are typically associated with abnormal uterine bleeding (AUB). Many polyps are asymptomatic. They can be detected as a result of infertility assessment, through finding of endometrial cells in cervical cytology, incidental endometrial sampling, pelvic imaging or hysteroscopy. 2

The diagnosis of endometrial polyps is a histological diagnosis that is based on the evaluation of the polyp after it is removed. The histological evaluation might rule out malignancy. In some women, endometrial polyps are diagnosed in the endometrial sample that has been taken to evaluate AUB. In such cases, polypectomy should be performed in the presence of an indication (with a view to relieving symptoms or ruling out malignancy) considering the possibility that the polyp may have not been completely removed with the endometrial sample. 3

Although endometrial polyps are usually benign, the risk of malignancy should still be kept in mind. American Association of Gynecologic Laparoscopists (AAGL) estimates that the prevalence of malignancy in endometrial polyps varies between 0% and 12.9% depending on the subgroups. 4

Hysteroscopic polypectomy is accepted as the standard treatment for an endometrial polyp. Polyps could be related to underlying endometrial pathologies. For that reason, both the polyp and the surrounding endometrium should be evaluated histologically. 5

Hysterectomy is a radical surgical option that eliminates the risk of polyp recurrence and malignancy potential. Hysterectomy is associated with a significantly higher risk and cost for the patient, and should only be considered after extensive patient counseling. 4

The aim of our study was to evaluate the definitive histopathological results of patients diagnosed with endometrial polyps in our clinic through endometrial sampling who underwent polypectomy by way of hysteroscopy or hysterectomy within one-year period after the diagnosis. We also aimed to determine the premalignancy/malignancy prevalence in patients with endometrial polyps and contribute to the literature by identifying factors that had an effect on the development of premalignancy/malignancy in these patients.

## Methods

In our retrospective study, 701 patients diagnosed with endometrial polyps through endometrial sampling were evaluated by examining the archives of the hospital information management system and patient files in Dicle University Faculty of Medicine, Gynecology and Obstetrics Clinic for the years between 2015 and 2020. Among these, 251 patients who were diagnosed with endometrial polyps through endometrial sampling and underwent polypectomy by way of hysteroscopy or hysterectomy within one-year period were included in the study. Ethical approval for our study was received from Dicle University Faculty of Medicine Ethics Committee with the decision dated 05.03.2020 and numbered 104.

Patients who were diagnosed with endometrial polyps through other diagnostic methods than endometrial sampling were not included in the study. Patients who were operated on in other healthcare organizations and those who were not operated on after endometrial sampling were excluded. Patients with endometrial sampling results not suggesting endometrial polyps and those with accompanying diagnoses such as malignancies or premalignant lesions were also excluded from the study.

The results of patients diagnosed with endometrial polyps through endometrial sampling were investigated one by one. The histopathological results of patients who underwent polypectomy by way of hysteroscopy or hysterectomy within one year were noted down. The results that were reported as hyperplasia without atypia were taken as benign. Then, the results were categorized as benign, premalignant (hyperplasia with atypia) and malignant. Premenopausal/postmenopausal status, indications for endometrial sampling, whether the patient was on hormone replacement therapy, size of the polyp (in centimeters) and demographic characteristics were noted down. Patients with amenorrhea for at least 12 months were considered postmenopausal.5 The sizes of polyps reported in the definitive histopathological results were noted in centimeters.

Endometrial sampling is a common procedure in our clinic. It is routinely performed in patients with AUB complaints; besides, those patients who do not have AUB but will receive hysterectomy in our clinic for other indications also undergo this procedure prior to hysterectomy. In this study, samples were taken from all the walls of the uterus in a blinded manner using a Karman aspirator under local anesthesia. Operation was recommended to the patients who were determined to have endometrial polyps as a result of the endometrial sampling, and the decision on operation was made together with the patient. Operative hysteroscopy was performed under spinal anesthesia in patients who underwent polypectomy by way of hysteroscopy. Saline infusion was used as an expander solution. After the cervical canal and the uterine cavity were checked, the polyp was resected by a resectoscope using bipolar coagulation electrode and plasma kinetic energy. Hysterectomy was performed under general anesthesia as abdominal, vaginal or laparoscopic hysterectomy. The specimens were placed in 10% formaldehyde solutions and sent to the Department of Pathology with the patient's clinical information.

SPSS 21 statistical software package was used for statistical analysis. Descriptive data were presented as mean, standard deviation, number and percentage. Kolmogorov Smirnov test was conducted to test the normality of the data. Chi square test was used to evaluate the categorical data. Student's t-test was used as a parametric test for the data that showed a normal distribution. Mann Whitney U test was used as a non-parametric test for data that did not show a normal distribution. A p value smaller than 0.05 was considered statistically significant.

## Results

The mean age of 251 patients that were included in the study was 45.4± 10.1 years. The mean size of the endometrial polyps (cm) was 1.5± 1.3 cm. 36 (14.3%) of the patients were postmenopausal. 235 patients (93.6%) had AUB complaints. It was determined that 181 of the patients (72.1%) underwent hysterectomy, and 7 patients (2.8%) had premalignant/malignant histopathological results (Table 1). Out of those seven patients with premalignant/malignant results, five had complex hyperplasia with atypia. From the patients with malignant results, one had endometrial stromal sarcoma, and another had a malignant mesenchymal tumor.

**Table 1:** Evaluation of demographic and clinical data of operated patients

	Mean±SD.(Min-Max)	
Age	45.4± 10.1 (22–78)	
Gravidity	5.5± 3.9 (0–16)	
Parity	4.8± 3.6 (0–15)	
Abortion	0.7± 1.1 (0–8)	
Number of living children	4.3± 3.2 (0–14)	
Endometrial polyp size (cm)	1.5± 1.3 (0.1–7.5)	
n=251		n (%)
Menopausal status	Premenopausal	215 (85.7%)
	Postmenopausal	36 (14.3%)
Abnormal uterine bleeding	Yes	235 (93.6%)
	No	16 (6.4%)
Hormone replacement therapy	Yes	130 (51.8%)
	No	121 (48.2%)
Indications for endometrial sampling	Abnormal uterine bleeding	235 (93.6%)
	Asymptomatic	16 (6.4%)
Hysterectomy	Yes	181 (72.1%)
	No	70 (27.9%)
Hysteroscopic polypectomy	Yes	70 (27.9%)
	No	181 (72.1%)
Histopathological results	Benign	244 (97.2%)
	Premalignant / malignant	7 (2.8%)

### Data are presented as mean ± SD and percent

Performing polypectomy by way of hysteroscopy in premenopausal women and hysterectomy in postmenopausal women was found to be significantly different (p< 0.05). Premenopausal women had a significantly higher rate of hormone replacement therapy use in comparison with postmenopausal women (p< 0.05). There was not a significant difference between the two groups in terms of histopathology results and the symptoms of the patients (AUB or asymptomatic) (p> 0.05) (Table 2).

**Table 2:** Comparison of data of premenopausal and postmenopausal patients

n= 251		Premenopausal	Postmenopausal	
		n (%)	n (%)	p
		n= 215	n= 36	
Abnormal uterine bleeding		204 (94.8%)	31 (86.1%)	0.091
Hormone replacement therapy		123 (57.2%)	7 (19.4%)	**0.000**
Hysteroscopic polypectomy		69 (32.1%)	1 (2.7%)	**0.001**
Hysterectomy		146 (67.9%)	35 (97.2%)	**0.000**
Indications for endometrial sampling	Abnormal uterine bleeding	204 (94.9%)	31 (86.1%)	0.138
	Asymptomatic	11 (5.1%)	5 (13.9%)	
	Benign	210 (97.7%)	34 (94.4%)	
Histopathological results	Premalignant / malignant	5 (2.3%)	2 (5.6%)	0.363

Data are presented as percent

Chi-square test

p<0.05 statistically significant (in bold)

When the data were assessed after the histopathological results were separated into two groups as benign and premalignant/malignant, gravidity, parity and number of living children were found to be significantly higher in the premalignant/malignant group in comparison with the benign group (p< 0.05). Performing hysterectomy in patients with premalignant/malignant results and polypectomy by way of hysteroscopy in patients with benign results was found to be significantly different (p< 0.05). However, there was not a statistically significant difference between the groups in terms of menopausal status, symptoms, whether the patient was on hormone replacement therapy or the size of endometrial polyps (p> 0.05) (Table 3).

**Table 3:** Evaluation of demographic and clinical data of benign and premalignant / malignant patients

		Benign		Premalignant/ Malignant n= 7		p
		n= 244				
		Mean±SD		Mean±SD		
Age		45.2± 10.0		52.4± 9.9		0.095
Gravidity		5.4± 3.9		9.2± 2.7		**0.009**
Parity		4.7± 3.6		8.1± 3.2		**0.018**
Abortion		0.7± 1.1		1.1± 1.4		0.321
Number of living children		4.2± 3.1		7.4± 2.6		**0.011**
Endometrial polyp size (cm)		1.5± 1.3		2.1± 2.4		0.605
n= 251		n	Yüzde %	n	Yüzde %	
Menopausal status	Premenopausal	210	(86.1%)	5	(71.4%)	
	Postmenopausal	34	(13.9%)	2	(28.6%)	0.363
Abnormal uterine bleeding	Yes	228	(93.4%)	7	(100%)	
	No	16	(6.6%)	0	(0%)	0.627
Hormone replacement therapy	Yes	126	(51.6%)	4	(57.1%)	
	No	118	(48.4%)	3	(42.9%)	0.540
Indications for endometrial sampling	Abnormal uterine bleeding	228	(93.4%)	7	(100%)	0.460
	Asymptomatic	16	(6.6%)	0	(0%)	
Hysterectomy	Yes	174	(71.3%)	7	(100%)	
	No	70	(28.7%)	0	(0%)	**0.031**
Hysteroscopic polypectomy	Yes	70	(28.7%)	0	(0%)	
	No	174	(71.3%)	7	(100%)	**0.031**

Data are presented as mean ± SD, Data are presented as percent

Mann Whitney U Testi

p<0.05 statistically significant (in bold)

## Discussion

There are still uncertainties surrounding the management of patients with endometrial polyps detected by endometrial sampling due to the possibility of current or future malignancies. In our study, we analyzed the definitive histopathological results of patients with endometrial polyps. We presented the premalignancy/malignancy prevalence and investigated the influential factors in order to contribute to the literature about the management of these patients. As a result of our study, we determined that postmenopausal status, presence of AUB complaints and endometrial polyp size did not have an effect on premalignancy/malignancy.

In our study, the diagnosis for endometrial polyps was made by endometrial sampling in all the patients that were included in the study. However, there are doubts in the literature surrounding the sufficiency of endometrial sampling in diagnosis. In a randomized controlled study, the endometrial sampling results of postmenopausal patients with AUB and a thickened endometrium were investigated. It was stated that of all patients with negative sampling results, 3% had undiagnosed endometrium cancer and 3% had endometrial hyperplasia with atypia accompanied by polyps.^3^ In another study that included 1050 patients with benign endometrial sampling results who underwent hysterectomy, 45 patients (4.2%) were preoperatively diagnosed with endometrial polyps. 13 patients (1.2%) had malignant definitive results, and it was stated that two of the patients with malignant results were operated on because of endometrial polyps.^6^ In a study conducted by Moradan et al., 163 patients underwent hysterectomy after endometrial sampling. The sensitivity of endometrial sampling in the diagnosis of hyperplasia and malignancy was found to be 62.5% and 83.3% respectively. 7 In another study, 51 patients who had endometrial polyps first underwent endometrial curettage and then hysteroscopy. As a result, it was concluded that endometrial curettage was insufficient to completely remove the endometrial polyps. 8 In their study, Salim et al. suggested that blind endometrial sampling was insufficient in diagnosing endometrial polyps and it should not be used as a diagnostic method.^2^ In agreement with the literature data, we also believe that endometrial sampling cannot be used to eliminate premalignancies and malignancies, and it cannot remove the polyps completely. For this reason, we are convinced that doctors should be careful and share these concerns with the patients and recommend an operation.

In a study, the results of 359 patients who were diagnosed with endometrial polyps using ultrasound and underwent polypectomy by way of hysteroscopy were analyzed. The mean age of the patients was reported as 53.0 ± 11.7 years. 178 patients (51.4 %) were postmenopausal, and 70.8% of the patients had AUB complaints. The number of patients with premalignant/malignant results was reported as 10.5 Another study was conducted on 256 patients with possible endometrial polyps in the ultrasound who underwent diagnostic hysteroscopy and polypectomy. In the study, the mean age was reported as 48.5±10.88 years. 34.4% of the patients were postmenopausal and 45.6% of the patients had AUB. Two patients were detected to have malignancies, and 33 patients had premalignant lesions (endometrial hyperplasia with or without atypia).9 In a different study on endometrial polyps, the carcinoma rate was found to be 4.7%, and the endometrial hyperplasia rate was 0.5%.^10^ In our study, the mean age of patients was 45.4 ± 10.1 years, and 36 patients (14.3%) were postmenopausal. 235 patients (93.6%) had AUB complaints, and 7 patients (2.8%) had premalignant/malignant histopathological results. Taking the literature data and our results into consideration and keeping in mind that endometrial polyps could be asymptomatic, it can be said that it is hard to determine their prevalence. For that reason, it can be seen that mean age and menopausal status were different in various studies. The reported premalignancy/malignancy prevalence of endometrial polyps varies in the literature. Depending on the subgroups of the studies, the prevalence varies between 0% and 12.9%.^4^ Therefore, it should be kept in mind that endometrial polyps may pose a risk of premalignancy and malignancy.

In our study, patients with endometrial polyps were separated into two groups as postmenopausal and premenopausal and then compared to each other. It was determined that the frequency of hormone replacement therapy and hysteroscopic polypectomy was significantly higher among premenopausal patients. In postmenopausal patients, on the other hand, hysterectomy was significantly more frequent. However, between the two groups of patients, there was not a significant difference in terms of AUB complaints, being asymptomatic or histopathological results. On the other hand, a previous study comparing premenopausal and postmenopausal women reported that there was a significant difference between the two groups in terms of mean polyp size and histopathological results. Postmenopausal women had significantly higher rates of premalignancy/ malignancy and larger sizes of endometrial polyps.^9^ In their study, Uglietti et al. reported that the malignancy prevalence of endometrial polyps was 2.73% and postmenopausal status and presence of symptoms increased the risk of malignancy in the polyps.11 Sasaki et al. stated that they found the premalignancy and malignancy prevalence to be 3.4% in their study. They determined that age over 60, presence of AUB and postmenopausal status were associated with the risk of malignancy in endometrial polyps. However, hormone replacement therapy, parity and endometrial polyp size were not associated with malignancy. 12 In a study by Namazov et al., postmenopausal patients that were asymptomatic underwent polypectomy by way of hysteroscopy. 2.33% of the patients had premalignant and malignant lesions. It was determined that the polyp size, use of hormone replacement therapy and age did not have an effect on premalignancy and malignancy. 13 Karakaş et al. conducted a study on patients who underwent polypectomy by way of hysteroscopy and were found to have endometrial polyps together with endometrial intraepithelial neoplasia as a result. It was stated that among these patients, those who were nulliparous and postmenopausal had a higher rate of concurrent endometrial cancer. 14 In our study, gravidity, parity and number of living children were found to be significantly higher in patients with premalignant/malignant results in comparison with patients with benign results. However, the polyp size, menopausal status, AUB complaints, age and use of hormone replacement therapy did not have an effect on premalignancy and malignancy. In our study, all of the patients with premalignancy/malignancy results were diagnosed after the hysterectomy. Hysterectomy was mostly performed on postmenopausal women. In a study by Elyashiv et al., it was stated that hysteroscopic polypectomy could not sufficiently eradicate premalignant and malignant endometrial lesions. They suggested that hysteroscopy should be put to use mostly in patients who want fertility preserving operations.^15^ In a study by Sheng et al., it was emphasized that the treatment in patients with endometrial polyps should be individualized and it should be decided together with the patient. In addition, it was suggested that hysteroscopic polypectomy should be performed in all postmenopausal and symptomatic patients. 16 Considering the literature data and our results, it is safe to state that factors affecting the malignancy and premalignancy risks in patients with endometrial polyps are still not clear. There are different studies reporting different results in the literature, which is attributable to the following: endometrial polyps can be seen in all age groups, there are patients who go undetected as they are asymptomatic, and some patients with endometrial polyps do not agree to the operation.

The retrospective nature of the study and the fact that we could not access some relevant data such as obesity and tamoxifen use that could affect the premalignancy/malignancy risks of endometrial polyps were the limitations of our study. One of the strengths of our study is that both endometrial sampling and definitive histopathologies of the patients were examined by pathologists of a tertiary hospital who were specialized in gynecology. Having asymptomatic patients and including patients who underwent hysteroscopy or hysterectomy in the study can also be considered some other strengths. In addition, the fact that endometrial polyps were diagnosed in each patient with the same histological method differs our study from others in the literature.

## Conclusion

the risk of premalignancy/malignancy in patients diagnosed with endometrial polyps as a result of endometrial sampling should be kept in mind. The patient's menopausal status, symptoms, polyp size and use of hormone replacement therapy should be taken into consideration when making the management plan, but these should not be the decisive factors.
